# WhatsApp Tele-Medicine – usage patterns and physicians views on the platform

**DOI:** 10.1186/s13584-021-00468-8

**Published:** 2021-06-01

**Authors:** Edward Barayev, Omri Shental, Dotan Yaari, Elchanan Zloczower, Itai Shemesh, Michael Shapiro, Elon Glassberg, Racheli Magnezi

**Affiliations:** 1 Department of Public Health and Health Systems Management Program, Bar Ilan University, 52900 Ramat Gan, Israel; 2 Israel Defense Forces Medical Corps, Tel Aviv, Israel

**Keywords:** WhatsApp, Consultations, Telemedicine, Social media

## Abstract

**Background:**

Telemedicine has become an integral part of health care delivery in recent years. One of the leading applications for this use is WhatsApp — a free smartphone application that allows instant messaging with pictures and videos. This study analyzed the emerging role of WhatsApp on reducing the need for referrals to medical specialists and to compare the views of physicians regarding WhatsApp consultations.

**Methods:**

A cross-sectional study based on an anonymous web-survey was conducted among PCPs and medical specialists working in the Israel Defense Forces Medical-Corps during September and October, 2019.

**Results:**

Of 201 participants, 153 were PCPs and 48 were medical specialists. 86.9 % of PCPs and 86.5 % of specialists used WhatsApp every day in professional settings. Added workload, potential breaching of patient confidentiality and lack of full documentation of consultations were the main concerns among physicians using the application. 60.7 % of PCPs and 95.7 % of specialists stated that these consultations have reduced the need for in-person appointments at least once a week.

**Conclusions:**

In times of COVID-19 that require social distancing, WhatsApp provides a simple, readily available platform for consultations between healthcare providers, even to the extent of rendering some in-person appointments unnecessary. Healthcare organizations should address the matters troubling healthcare providers, mainly patient confidentiality and lack of documentation in patients’ medical records, while providing adequate compensation for those providing the service during and after work hours.

## Background

Technological developments in the recent decade have dramatically changed the way we live, work and communicate. Mobile phone penetration in developed countries is over 90 %, and over 65 % of the US population owns a smartphone [1]. Among healthcare providers, these rates are even higher [2, 3]. According to recent surveys, Israel is ranked among the top 10 countries worldwide in both internet use and smartphone ownership, with 76 % of smartphone owners reporting frequent use of social network applications [4].

Prior to these technological developments, to consult with a medical specialist, the patient had to schedule a physical appointment. This resulted in long waiting times, loss of work days and suboptimal patient experience. Furthermore, when a primary care physician (PCP) needed to consult with a specialist, a formal referral was usually necessary.

Recently, healthcare professionals have been seeking alternatives to this cumbersome process, especially by using telemedicine [5, 6]. Today, both PCPs and patients can reach a medical specialist directly through social media or instant messaging applications, potentially reducing time to diagnosis as well as the time to subsequent treatment [7].

Recent publications have studied the role of social media applications as a communication method between PCPs, medical specialists and patients [7–9]. The leading social media application for this use is WhatsApp (WhatsApp Inc., Mountain View, CA, USA), a free smartphone application that allows instant messaging, including media content such as photographs, voice messages and videos [8, 10]. Previous studies have explored the benefits and shortcomings of professional WhatsApp use in healthcare organizations [8, 10–12]. Recent studies have shown that communications between specialists and non-specialists using WhatsApp, among other telemedicine applications, achieved high rates of diagnosis without the need for patient referrals, alongside high patient satisfaction [13–15]. It is important to note that WhatsApp is not the only applications available; as a recently published survey among 802 providers from 56 countries has shown, Zoom, GoToMeeting, Blu Jeans, Skype, FaceTime, Google, Microsoft Teams, WeChat and WhatsApp were all used, with the last two accounting for the majority of consultations [16].

The coronavirus pandemic (COVID-19) has caused a significant shift towards tele-medicine consultations among many health care providers all across the globe [17, 18]. As social distancing became a substantial tool for lowering the risk of patients and providers’ exposure to COVID-19, consultations with specialists using WhatsApp becomes more relevant than ever [19].

This study set to analyze the role of WhatsApp on the work interface between PCPs and medical specialists, whether consultations conducted using WhatsApp have indeed reduced the need for referring to specialists. Furthermore, we investigated the personal views of PCPs and medical specialists regarding the advantages and disadvantages of WhatsApp consultations.

## Methods

### Study design and population

This cross-sectional study was conducted during September and October 2019 among PCPs and medical specialists in the Israel Defense Forces (IDF) Medical Corps, using a web-based survey.

The IDF medical corps is the sole provider of health services to all active duty soldiers, consisting mostly of primary care physicians (PCPs) distributed across the entire country – in both dense and rural areas. Medical specialists are also employed by the IDF, mostly in military medical centers, situated in large cities and in proximity to civilian, tertiary medical centers.

Inclusion criteria:

1. PCPs or medical specialist working in the IDF.
2. Registered physician in the state of Israel.
3. Valid email contact.

We distributed a link to the questionnaire using both e-mail and phone messages, explaining the objective of the study and inviting potential participants to answer the voluntary, anonymous questionnaire.

E-mail and phone messages were sent 3 times (every 2 weeks) to all potential participants during the study period, starting September 6, 2019.

### Ethical considerations

 The study was deemed exempt from obtaining institutional review board approval by the Israel Defense Forces Medical Corps Institutional Review Board, as no patient information was accessed, and the questionnaire was completely anonymous and distributed only among medical staff. Invitations to complete the survey included the main study objectives, as well as the right not to participate by simply not filling the questionnaire.

### Study instrument

The study used a self-administered survey using Google Forms (Google LLC, Mountain View, CA, USA), developed by the authors based on previously published questionnaires which were forwarded from the original authors, translated and validated using an expert committee consisting of 2 specialists, 2 primary care physicians, 2 translators, a statistician and the authors of this study [2, 8]. The questionnaire was administered in Hebrew, and included questions regarding use of WhatsApp, consultations between physicians, and perceived benefits and disadvantages of using WhatsApp. Multiple submissions were prevented by limiting only one response per person using Google Forms settings. A preliminary pilot testing was first conducted among 20 respondents (10 PCPs and 10 specialists), which were asked to elaborate regarding the questions and their responses. Consistency of the questionnaire was validated prior to distributing the survey, by analyzing the answers of these 20 respondents, representative of the study population (Cronbach’s alpha = 0.919). No significant changes were introduced following the pilot.

The questionnaire was divided into 4 sections. The first consisted of 4 questions that quantified use of social networking sites (e.g. Facebook) and WhatsApp in both personal and professional settings. Usage characteristics were categorized as “low” (not at all, few times a week), “medium” (at least once daily) or “high” (at least every hour, multiple times per hour) as indicators of frequency of use.

The second section included 3 questions regarding the frequency of medical consultations — between colleagues, between PCPs and medical specialists, and using medical applications. These questions were divided into “Achieving a diagnosis”, “Workup selection”, “for consulting on treatment/dosage (Treatment selection)” and “Staying updated with current medical knowledge”. Options were consistent with those described in the first section of the questionnaire.

The third section included 3 questions regarding the best perceived consultation tools for physicians, whether any of these tools led to a reduction in referrals to medical specialists, and what were the most frequently consulted medical specialties.

The fourth section included 5 questions regarding the perceived benefits and disadvantages of using WhatsApp. Benefits included were “Media sharing”, “Rapid consultation (faster response compared to phone call or email)”, “Option to answer when comfortable, “Ability to refine a consultation before submitting it”, and 2 possible advantages of WhatsApp groups – “Venting with colleagues”, and “Keeping informed of new directives and updates”. Potential disadvantages included: “Increased workload during work hours”, “Consultations outside of work hours”, “Breaching patient confidentiality”, “Failure to document in medical records” and “Invading physician’s personal space”. Participants were also directly asked whether most WhatsApp consultations were during working hours, and whether they get requests after 20:00, during weekend/holidays, on leave days, or when abroad.

### Statistical Analysis

Data collected were analyzed using SPSS, version 24.0 (SPSS Inc., Chicago, IL, USA). Descriptive statistics were calculated for all variables, and were shown as frequencies and percentages. Student’s t-test was used to compare usage characteristics between PCPs and medical specialists. Paired samples t-test was used to compare variables within each participant. Chi-square test was used to compare the categorical variables between the 2 study groups of PCPs and medical specialists. A p-value < 0.05 was considered statistically significant.

## Results

### Participant Characteristics

**Fig. 1:**
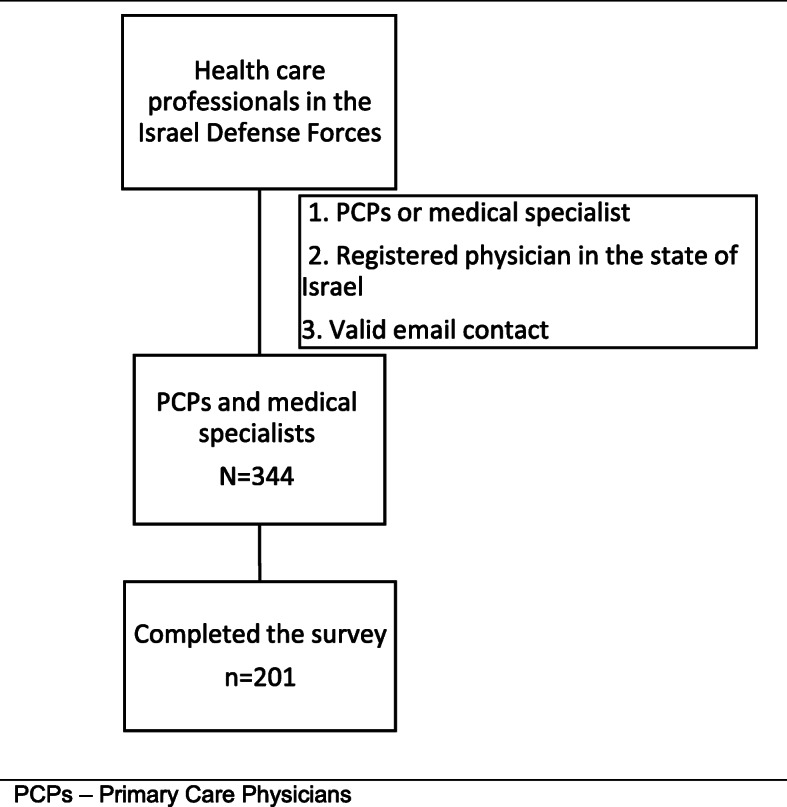
Flow of participants through the study

Among 344 physicians who matched the inclusion criteria, 201 (59 %) responded and completed the online survey (Fig. 1). Of these, 153 were primary care physicians (PCPs) and 48 were medical specialists. Mean age of the 201 respondents was 31 (SD 7, range 26–59), 136 were men (67.7 %), with mean work experience of 5 years (SD 6.2, range 0–40). Demographic data of study participants are shown in Table 1.

**Table 1:** Participant characteristics

Characteristic	PCPs (*n* = 153) N (%)	Medical Specialists (*n* = 48) N (%)	*P*-value
Age in years, mean (SD)	31.31 (4.94)	42.9 (5.003)	< 0.001
**Sex**			0.051
Men	98 (64.1)	38 (79.2)	
Women	55 (35.9)	10 (20.8)	
Practice experience in years, mean (SD)	5.24 (5.55)	11.88 (6.01)	< 0.001

*PCPs* Primary Care Physicians; *SD* standard deviation

### App Usage Characteristics

As can be seen in Table 2, over 85 % of PCPs and medical specialists reported professional use of WhatsApp at least once a day (medium and high frequencies). Over 87.5 % of both groups reported low frequency of professional use of Facebook.

**Table 2:** Usage characteristics between primary care physicians (PCPs) and medical specialists

Characteristic		PCPs (n = 153) N (%)				Medical Specialists (n = 48) N (%)				*P*- value
		Low^a^		Medium	High	Low		Medium	High	
**Facebook use**										
Personal		63 (41.2)		78 (51)	12 (7.8)	25 (52.1)		17 (35.4)	6 (12.5)	0.377
Professional		145 (94.8)		8 (5.2)	0 (0)	42 (87.5)		4 (8.3)	2 (4.2)	0.065
**WhatsApp use**										
Personal		1 (0.7)		27 (17.6)	125 (81.7)	0 (0)		9 (18.8)	39 (81.2)	0.923
Professional		20 (13.1)		45 (29.4)	88 (57.5)	7 (14.5)		14 (29.2)	27 (56.3)	0.986
**Medical app use (Medscape, Uptodate, etc.)**										
Achieving diagnosis		69 (45.1)		63 (41.2)	21 (13.7)	30 (62.5)		16 (33.3)	2 (4.2)	.*05*
Workup selection		70 (45.8)		65 (42.5)	18 (11.8)	36 (75)		10 (20.8)	2 (4.2)	*< 0.001*
Treatment selection		34 (22.2)		90 (58.8)	29 (19)	25 (52.1)		19 (39.6)	4 (8.3)	.*001*
Staying current with medical knowledge		88 (57.5)		52 (34)	13 (8.5)	26 (54.2)		18 (37.5)	4 (8.3)	0.992
**Consulting colleagues using WhatsApp**										
Achieving diagnosis		68 (44.4)		70 (45.8)	15 (9.8)	29 (60.4)		15 (31.3)	4 (8.3)	0.242
Workup selection		90 (58.8)		52 (34)	11 (7.2)	37 (77.0)		8 (16.7)	6 (6.3)	0.314
Treatment selection		83 (54.2)		56 (36.6)	14 (9.2)	30 (62.5)		16 (33.3)	2 (4.2)	0.434
Staying current with medical knowledge		111 (72.5)		33 (21.6)	9 (5.9)	35 (72.9)		10 (20.8)	3 (6.3)	0.110
**Best tool for consultations**^**b**^										
Consulting colleagues using WhatsApp		55 (35.9)				10 (20.8)				0.051
Consulting specialists using WhatsApp		84 (54.9)				24 (50)				0.552
Consulting specialists via a phone call		48 (31.4)				25 (52.1)				.*009*
Online clinical resources		85 (55.6)				10 (20.8)				*< 0.001*
In-person appointment with specialist		23 (15)				17 (35.4)				0.002

^a^ “low” = Not at all/ few times a week; “medium” = at least once daily; “high” = at least every hour.

^b^ Each physician could select up to 2 consultation tools.

The most frequently consulted specialties by PCPs were dermatology (85.6 %), orthopedic surgery (55.3 %), ophthalmology (21.8 %), and otolaryngology (17.1 %).

To achieve a diagnosis, 55.4 % of PCPs used WhatsApp at least once a day, compared with 39.6 % of specialists (*p* = 0.242). For workup selection, only 41.2 % of PCPs and 23 % of medical specialists reported frequent use of WhatsApp (*p* = 0.314). For treatment selection, similar WhatsApp usage was described among PCPs (45.8 %), with slightly higher usage among medical specialists (37.5 %; *p* = 0.434).

Similar proportions were reported among PCPs for using WhatsApp and other medical apps (Medscape, UpToDate, etc.) in the diagnostic process (54.9 % vs. 55.6 %, p = 0.546, paired samples t-test). However, for both workup and treatment selection, PCPs preferred using other medical apps over WhatsApp (77.8 % vs. 45.8 %, *p* < 0.001, and 54.3 % vs. 41.2 %, *p* < 0.001, paired samples t-test), respectively.

Both PCPs and medical specialists reported low use of WhatsApp for the purpose of staying current with medical knowledge (72.5 and 72.9 %, *p* = 0.110).

### Patient Referrals and Consultations

When asked for the best tool for consultation among medical staff, major differences were noted regarding the use of online resources and telephone consultations. Online resources were the most favored tool among PCPs, compared with medical specialists (55.6 % vs. 20.8 %, *p* < 0.001), while medical specialists preferred consultations via telephone (32.4 % vs. 52.1 %, *p* = 0.009).

**Fig. 2:**
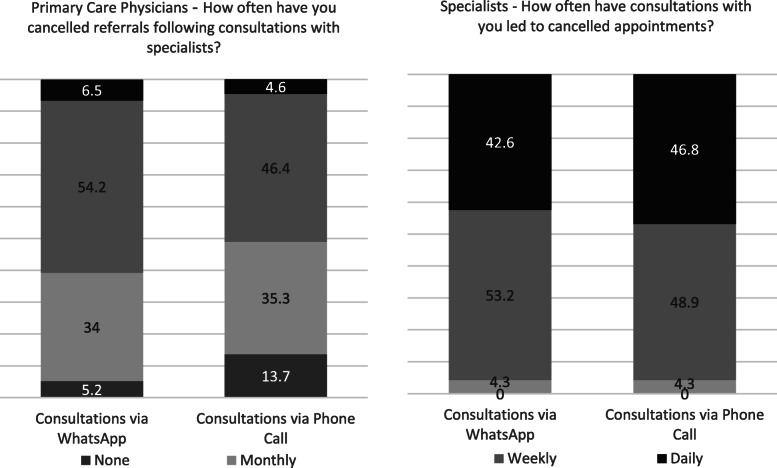
Patient referral cancellations following WhatsApp and phone call consultations

Cancellations of patient referrals to medical specialists or rendering such referrals unnecessary following WhatsApp and phone call consultations were examined from the perspectives of both PCPs and medical specialists. PCPs reported higher rates of cancellations on a weekly basis following WhatsApp usage compared with phone calls (60.7 % vs. 51 %, p = 0.001, paired samples t-test). Medical specialists reported similar proportions of cancellations using WhatsApp and phone calls (95.7 % for both modalities on a weekly basis, *p* = 0.183, paired samples t-test) **(**Fig. 2**)**.

### Perceived Advantages and Disadvantages

Both groups rated WhatsApp as a highly effective tool for improving healthcare delivery. However, on a scale of 1 to 6, PCPs considered WhatsApp more beneficial than did medical specialists (5.22 vs. 4.71, respectively; *p* = 0.005). Both groups rated media sharing as the main advantage of WhatsApp (88.9 and 81.3 %, *p* = 0.169). Slight difference was observed regarding the perceived benefit of rapid consultation, rated higher by PCPs (69.3 %) than by specialists (50 %, *p* = 0.015); Table 3).

**Table 3:** Comparison of perceived advantages and disadvantages of WhatsApp consultations between primary care physicians and medical specialists.

	Primary Care physicians (*n* = 153), N (%)	Medical Specialists (*n* = 48), N (%)	*P*-value
How much has WhatsApp improved healthcare delivery?^a^ Mean (SD)	5.22 (0.85)	4.71 (1.11)	.*005*
**What are the main advantages of WhatsApp?**			
Media sharing	136 (88.9)	39 (81.3)	0.169
Rapid counseling	106 (69.3)	24 (50)	.*015*
Answering when comfortable	69 (45.1)	28 (58.3)	0.109
Ventilating with colleagues	44 (28.8)	8 (16.7)	0.095
Keeping informed on medical updates	36 (23.5)	12 (25)	0.835
Refining content of consultation	36 (23.5)	12 (25)	0.835
How much has WhatsApp harmed the clinical setting?^a^ Mean (SD)	1.94 (0.89)	2.17 (1.08)	0.148
**What are the main disadvantages of WhatsApp?**			
Consultations outside of work hours	105 (68.6)	29 (60.4)	0.292
Increased workload during work hours	110 (71.9)	27 (56.3)	.*042*
Failure to document in medical records	77 (50.3)	28 (58.3)	0.333
Breaching patient confidentiality	41 (26.8)	18 (37.5)	0.155
Invading physician’s personal space	32 (20.9)	6 (12.5)	0.194
**WhatsApp consultation characteristics**			
Most consultations are during work hours	115 (75.2)	42 (87.5)	0.071
Frequent consultations after 20:00	128 (83.7)	37 (77.1)	0.300
Frequent consultations on weekends / holidays	121 (79.1)	32 (66.7)	0.078
Frequent consultations on sick leave / vacations	136 (88.9)	45 (93.8)	0.326
Frequent consultations when abroad	109 (71.2)	43 (89.6)	.*010*

^a^ On a scale of 1–6, 6 being the highest

On a scale of 1 to 6, both groups rated WhatsApp’s potential for harming the clinical setting as relatively low (1.94 vs. 2.17, p = 0.148). PCPs rated increased workload during working hours as the main disadvantage, significantly higher than medical specialists did (71.9 % vs. 56.3 %, *p* = 0.042). Both groups rated consultations after working hours (68.6 and 60.4 %, *p* = 0.292) and lack of documentation in official medical records as the main pitfalls of using WhatsApp (50.3 and 58.3 %, respectively; *p* = 0.333).

Most physicians in both groups reported that even though most consultations were during working hours, they were also frequently consulted after 20:00, during weekends, holidays, sick leaves and vacations. It is worth noting that medical specialists reported significantly higher consultations when abroad than PCPs did (71.2 % vs. 89.6 %, respectively; *p* = 0.010).

## Discussion

WhatsApp is a simple, readily available tool used daily by most PCPs for professional purposes, that allows the managing physician to consult with his peers, and easily provide tele-medicine consultations for his patients. As a result, it improves healthcare delivery by preventing excessive patient referrals, as over 95 % of specialists in our study state. In times of COVID-19, when many healthcare facilities switch to various forms of tele-medicine consultations, WhatsApp becomes more relevant than ever.

The IDF Medical-Corps issued their official guidelines in 2017, formally addressing the issue of medical consultations via WhatsApp and other digital applications. In these guidelines, special focus was given to the topics of patient confidentiality and documentation of medical consultations. For instance, no identifiable information is allowed to be delivered using non-secured methods such as WhatsApp, and if images are to be sent, then extra caution has to be taken in order to avoid sending identifying photos (e.g. eyes, face, identity number, etc.). The consulting PCP holds the responsibility for manually documenting the consultation in the patients’ medical records.

Another aspect that was addressed by the IDF-MC is the compensation provided for this service. Providing consultations for primary care physicians by specialists has been defined as part of the responsibilities of several consulting specialists in the army. Although no extra payment is currently provided for the service, defined time slots have been allocated in their weekly schedule for such tasks. In the future, WhatsApp consultations will be compensated in a similar fashion to other tele-medicine consultations.

Tele-dermatology was previously described as especially effective in rural clinics in the IDF, even before the era of smartphones, using e-mails. As Klaz et al. [20] showed in their study, tele-diagnosis was possible for 95 % of referrals. Satisfaction was rated especially high in rural clinics, both by patients and physicians. As the quality and availability of mobile phone cameras continue to improve, we believe this could prove to be of real value to PCPs in remote clinics, potentially replacing long journeys for patients to central secondary care clinics.

 As our data show, physicians provided explanations for preferring WhatsApp as a means of communication between physicians: (1) Allowing sharing of videos and images; (2) the ability to edit messages before sending them, in contrast to phone consultations where one might not always be ready for questions asked by the specialist; and (3) the ability to choose to respond at a convenient time. It is interesting to note that although the majority of specialists selected this last response, one of the main advantages noted by PCPs was the rapid response to consultations, thus somehow indicating that low availability was not a serious issue raised by PCPs.

Similar to previously published articles, our data suggest that the majority of WhatsApp consultations are in the fields of dermatology and orthopedic surgery [21–24]. This can largely be attributed to the ability to share high resolution photos of obviously visible lesions and other pathologies using the physician’s smartphone camera.

It is important to note that specialists in our study ranked WhatsApp lower when asked how much it has improved patient care (4.71 vs. 5.22 on a scale of 1–6, Table 2). This could perhaps reflect the specialists’ longer experience with other consultation platforms, as well as reduced need for consultations with their peers.

Despite that, almost all specialists in our survey agreed that WhatsApp consultations have replaced referrals of patients at least once a week. Previous studies have stated even higher rates [25, 26], potentially making WhatsApp also a cost-effective tool, saving time and resources for both physicians and patients by avoiding unnecessary time and travel. Recent studies in times of COVID-19 also suggest WhatsApp as a viable alternative for in-person appointments with specialists in various medical fields [23, 27].

The added workload both during and after work hours was noted as the biggest disadvantage of WhatsApp. WhatsApp can certainly be a doubled edged sword in that respect, as work can intertwine with our personal time, potentially leading to burnout. We believe an appropriate financial compensation method for medical specialists providing consultations through WhatsApp should be instituted in organizations, similar to other platforms of telemedicine.

Other potential risks many physicians noted were breaching patient confidentiality and lack of full documentation of consultations in patients’ medical records. As most healthcare organizations do not directly address these concerns, they could pose legal issues and limit usage among physicians and patients [28, 29]. An important matter when addressing patient confidentiality is that although WhatsApp claims end-to-end encryption in their service, it is not HIPAA (Health Insurance Portability and Accountability Act) compliant, as WhatsApp does not have mandatory password protection, and messages may be displayed on locked phone screens, in addition to being incorporated into the user’s photo library on their phone, which may pose as a privacy issue as well. Also, as messages and attachments sent through WhatsApp are only backed up locally, that could risk later documentation.

Our study population consisted mostly of young PCPs serving in the Israel Defense Forces, frequently stationed at distant, rural locations. We believe our results show WhatsApp can act as a tool to bridge the gap between the center and periphery in Israel, alongside longer-term plans such as building medical schools and using financial incentives in order to attract physicians to work in the periphery [30, 31].

Our study had several limitations. Selection bias was caused by using a convenience sample of physicians in one organization, employed generally in remote clinics. Results could differ in other organizations, especially in large clinics that employ both PCPs and medical specialists. However, we believe our findings are relevant for all healthcare services providing primary care in remote clinics, distant from tertiary medical centers. Also, most of the physicians were young, which could partially explain the high usage of social media, explored in this study. Lastly, in such a rapidly evolving reality, by the time this paper is published, there will probably be more studies available regarding the topic of WhatsApp and telehealth delivery. Further studies are required to assess the usage of social media in other healthcare settings and providers, and the impact on providing fast and accurate medical diagnoses and treatment.

## Conclusions

WhatsApp provides a free, widely spread platform for tele-medicine, even to the extent of rendering some in-person appointments unnecessary. It can help in bringing rapid specialist consultations to distal rural areas, and to patients in risk groups for COVID-19 infection, who should minimize their exposure to other patients and stick to social distancing guidelines. Healthcare organizations need to directly address the possible weaknesses troubling healthcare providers, mainly patient confidentiality and lack of documentation in medical records, in order to minimize inappropriate use of social media applications, such as WhatsApp by healthcare providers. An appropriate financial compensation system for these physician consultations should also be instituted in healthcare organizations, similar to that for other tele-medicine platforms.

## Data Availability

The datasets used and/or analyzed during the current study are available from the corresponding author on reasonable request.
